# “Biofeedback-based return to sport”: individualization through objective assessments

**DOI:** 10.3389/fphys.2023.1185556

**Published:** 2023-06-12

**Authors:** Antonis Ekizos, Alessandro Santuz

**Affiliations:** ^1^ Olympic Training Center of Berlin, Berlin, Germany; ^2^ Max-Delbrück-Center for Molecular Medicine in the Helmholtz Association (MDC), Berlin, Germany

**Keywords:** elite sport, locomotion, customization, biomechanical measurements, functional testing, biofeedback, athletics, neuromuscular coordination

## Abstract

Elite athletes are regularly exposed to high and repetitive mechanical stresses and impacts, resulting in high injury rates. The consequences of injury can range from time lost from training and competition to chronic physical and psychological burden, with no guarantee that the athlete will return to preinjury levels of sport activity and performance. Prominent predictors include load management and previous injury, highlighting the importance of the postinjury period for effective return to sport (RTS). Currently, there is conflicting information on how to choose and assess the best reentry strategy. Treating RTS as a continuum, with controlled progression of training load and complexity, seems to provide benefits in this process. Furthermore, objectivity has been identified as a critical factor in improving the effectiveness of RTS. We propose that assessments derived from biomechanical measurements in functional settings can provide the objectivity needed for regular biofeedback cycles. These cycles should aim to identify weaknesses, customize the load, and inform on the status of RTS progress. This approach emphasizes individualization as the primary determinant of RTS and provides a solid foundation for achieving it.

## 1 Introduction

Elite athletes are exposed to repeated mechanical stress and high levels of impact on their bodies, both during training and competition. One of the highest injury rates in professional sport is encountered in elite athletics in particular. A recent survey of retired Olympians ranks athletics as one of the five sports with the highest lifetime prevalence of injury (Palmer et al., 2021). Similarly, a 1-year prospective study on an elite cohort in athletics that included all injuries revealed a staggering annual injury incidence of 68%, half of which (51%) were classified as severe (i.e., causing a period of absence from normal training for more than 3 weeks) (Jacobsson et al., 2013). Another study reported a similar incident rate (65%) (Edouard et al., 2022). Injuries in athletics often occur during competition. Between 2007 and 2012, one in every twelve registered athletes suffered an injury (categorized as resulting to time-loss) at international athletics championships (Alonso et al., 2012; Feddermann-Demont et al., 2014). Further, these injuries take a significant amount of time to heal, often sidelining athletes for weeks or even months and adversely affect the careers of elite athletes (Palmer et al., 2021).

Poor load management is often cited as the culprit of high injury rates, yet previous injury is also an important predictor (Jacobsson et al., 2013; Soligard et al., 2016). While the first received a lot of scientific attention in recent years (Kuipers and Keizer, 1988; Meeuwisse, 1994; Drew and Finch, 2016; Schwellnus et al., 2016; Soligard et al., 2016; Jones et al., 2017; Eckard et al., 2018), the latter is inherently more complicated to objectively assess and quantify, for instance due to different locations of injuries, variegated causality mechanisms and rehabilitation practices, and so on. After an injury, the athlete is eventually called to reenter the sporting activity and the decision-making process at this stage is extremely strenuous. In fact, in the 24 months after anterior cruciate ligament reconstruction, athletes had a greater risk to suffer a subsequent injury compared with young athletes without a history of anterior cruciate ligament injuries (Paterno et al., 2014). Consensus regarding the period after the typical medical treatment is scarce and generalized advice is typically convoluted (Ardern et al., 2016a). To make matters more complex, there is strong evidence for heterogeneity in the responsiveness to physical activity (Bouchard and Rankinen, 2001), which can be attributed to innate and acquired characteristics simultaneously (Ross et al., 2019). This interindividual variability in response to a prescribed post-injury training program necessitates a way to track these responses and raises questions about existing methods.

In addition to the difficulties in choosing and assessing the best reentry strategy, there is no guarantee that the athlete will return to preinjury levels of sport activity and performance. For instance, following anterior cruciate ligament reconstruction surgery, 81% of people returned to any sport, 65% returned to their preinjury level of sport and only 55% returned to competitive level sport after surgery (Ardern et al., 2014). Return to the preinjury level of sport following the same procedure is 83% among elite athletes (Lai et al., 2018). The consequences can also be chronic and/or affect mental health. One-third of a high sample survey on retired elite athletes reported pain and functional limitations that were present up to the time of survey due to those injuries, while depression was more prevalent in those who have sustained a significant injury (Palmer et al., 2021). Thus, it is essential that these decisions are based on the best available evidence and are made in the athlete’s best interests. Not only do these decisions affect an athlete’s ability to compete at the highest levels, but they also have significant implications for an athlete’s long-term health and wellbeing.

The purpose of this article is to 1) give an overview of the injury landscape in elite sports, 2) present the current state of the return to sport (RTS) complexity, 3) examine different frameworks of RTS, 4) offer perspectives on the increasingly important role of objective assessments in the field and v) consider future directions. The article skews towards athletics, but the considerations presented thereafter might be relevant to most elite sports.

## 2 Location and mechanisms of injury

In a survey sample of 3,357 retired Olympians of all sports, two-thirds of them reported significant injuries in their careers, with the knee, lumbar spine, and shoulder/clavicle being the most commonly injured areas (Palmer et al., 2021). The anatomical site of injury (e.g., bone stress injuries) can influence rehabilitation timelines and the risk of complication (Hoenig et al., 2023). A study that examined injuries during the 2016 summer Olympics found that the most commonly injured anatomical locations were, in order of more to less prevalent, the knee, thigh, ankle, face and lower leg (Soligard et al., 2017). In elite athletics, most of the time-loss injuries in competition (2007–2012) affected the lower extremity (87.1%), followed by the upper extremity (6.1%) and the trunk (5.9%). The thigh was the most commonly injured body part (34.5%), followed by the lower leg (14.6%), foot (9.8%), and knee (9.6%) (Feddermann-Demont et al., 2014).

In general, the causes behind injuries in elite athletes are multifactorial and often include a combination of intrinsic (e.g., muscle weakness or previous injuries) and extrinsic (e.g., equipment, field conditions, training volume, *etc.*) factors (Meeuwisse, 1994; Meeuwisse et al., 2007). However, adaptations occurring within the context of a specific sport alter the injury risk and affect the aetiology in a dynamic, recursive fashion (Meeuwisse et al., 2007). Overtraining and overuse have long been identified as a major drive for injury in elite athletes (Kuipers and Keizer, 1988; Meeuwisse, 1994; Schwellnus et al., 2016; Jones et al., 2017; Eckard et al., 2018), especially in disciplines of the summer Olympics (Palmer et al., 2021). In a prospective report from British elite athletics, the proportion of overuse injuries was approximately 70% of all injuries, while in a study of Swedish elite athletics, the percentage was around 96% (Jacobsson et al., 2013; Kelly et al., 2022). The discrepancy may be explained by differences in overuse and acute injury definitions in the two studies. Inconsistencies within the literature regarding the use of various terminologies such as training load, fatigue, injury and illness are limiting our ability to develop a complete model of association (Jones et al., 2017). Here, we use the term “load” as “the sport and non-sport burden as a stimulus that is applied to a human biological system” (Soligard et al., 2016). There is strong evidence for a load-injury relationship in tactical and elite athlete populations, with higher emphasis on the overall amount of load and less on its frequency of application (Eckard et al., 2018). Acute changes in the training program have also been shown to be a common mechanism of injury (Jones et al., 2017). Predicting injury events would be of immense value in elite sports, and several indicators have been examined. The most prominent include the self-reported perceived exertion questionnaire and the “*acute to chronic load ratio”*. With both methods it is possible to obtain good association and, thus, prediction of injury risk (Gabbett et al., 2016; Eckard et al., 2018).

Previous injuries are also an important predictor of follow-up injuries, highlighting the importance of the rehabilitation and RTS periods, since it has been shown that the variability of RTS duration and responsiveness to therapy can affect possible reinjury risk (Meeuwisse, 1994; Meeuwisse et al., 2007). During the first 9 months after anterior cruciate ligament reconstruction surgery, a later RTS has shown to be associated with a lower reinjury rate (Grindem et al., 2016). Remarkably, for every 1-month delay in return to sport, the reinjury rate was reduced by 51% (Grindem et al., 2016). However, after the 9-month mark, time was no longer a predictor of injury (Grindem et al., 2016) making it unusable for determining readiness to RTS. Further, we know that the individual’s psychology and the sociocultural substrate affect injury risk, response, and recovery and both should also be accounted for (Galambos et al., 2005; Wiese-Bjornstal, 2010). These aspects add to the overall complexity and make it difficult to build appropriate predictive models.

## 3 Return to sport: definitions and consensus

Concepts such as return to preinjury levels in terms of training and competition participation after injury are naturally difficult to consistently define. The terms RTS, return to play, return to performance and others have been often used interchangeably to describe several stages of reconditioning following injury (Doege et al., 2021). Most studies use the term RTS when the athlete competes again in a game, while other variants refer to it as returning to training and/or explicitly defined competition levels and objectives (Doege et al., 2021). Unfortunately, this lack of consensus can lead to inaccurate comparisons, making it difficult to manage patient expectations and recovery (Kyritsis et al., 2016; Wiggins et al., 2016; Doege et al., 2021).

Returning to preinjury levels of performance is the primary measure of success from the athlete’s standpoint (Palmer et al., 2021). Historically, RTS has often been considered a single end point, reached when the athlete returns to competition or game. However, it is recently recognized as a more complex and gradual process starting when the athlete return to training and up to the point of return to previous levels of performance (Ardern et al., 2016a; Snyders et al., 2023). This can be seen as a continuum that is paralleled with recovery and rehabilitation (Ardern et al., 2016a), and in which the effects of injury and their alleviation should be actively incorporated to the ongoing training.

A recent consensus statement described the RTS continuum and identified three elements or stages: 1) return to participation, such as modified training, including ongoing rehabilitation, but not been able to return to competitive sport; 2) RTS, characterized by returning back to the same sport, but not returning back to previous levels of performance; 3) return to performance, describing the resumption of sport performing at or above his or her preinjury level (Ardern et al., 2016a). A similar, slightly different model suggested four distinct phases of RTS progression including: 1) on-field rehabilitation; 2) return to training; 3) return to competitive match play; 4) return to performance (Buckthorpe et al., 2019). Recognizing RTS as a continuum provides several advantages, primarily by allowing the RTS stakeholders to better understand and frame this process correctly. Identifying and achieving the milestones described above can then be used as is or adapted to suit individual cases and needs. Using the term RTS in the context of a continuum, or “*RTS continuum”* as an umbrella term, could provide much needed consistency in the literature. Here, we have chosen the first option, “RTS”, in line with recent literature (Ardern et al., 2016a; Doege et al., 2021).

## 4 Return to sport frameworks

RTS is influenced by a multitude of physical and non-physical factors. Conceptualisation of the connectivity between different factors are one important way to empower and inform the team around the athlete of what influences RTS (Ardern et al., 2016a). The development of such frameworks helps to understand and guide the RTS process by promoting consistency in decision making and minimizing risk.

The *three-step decision-based model* was introduced in 2010 (Creighton et al., 2010) and improved upon later, with the *Strategic Assessment of Risk and Risk Tolerance (StARRT)* (Shrier, 2015). This framework helps to estimate the risks of different short-term and long-term outcomes associated with RTS. It introduces generic standardizations and factors that should be considered by the RTS decision maker for risk assessment. The *biopsychosocial model* includes biological, psychological, and social factors and has been repurposed for the context of sport injury (Wiese-Bjornstal et al., 1998; Ardern et al., 2016b). For instance, after sustaining an injury, 75.5% of retired elite athletes indicated that they put pressure on themselves to return to sport as quickly as possible, followed by pressure from coaches (33.6%), sport governing body (15.5%) and teammates (13.6%) (Palmer et al., 2021). This can influence treatment decisions and outcomes after injury and therefore affect the RTS process. The *Goldilocks approach* uses the “*acute to chronic load ratio”* to permit a quantification of an athlete’s risk of subsequent injury (Blanch and Gabbett, 2016; Gabbett et al., 2016). The quintessence is that gradual increases in overall fitness (chronic load) should be sufficient to overcome sudden fatigue demands (acute load). This ratio can be a useful tool in planning load progressions in order to optimally prepare athletes for competition, minimize the risk of injury, as well as, during the RTS continuum. Another consideration is possible neuroplastic disruptions (e.g., after anterior cruciate ligament rupture) after injury, which might affect motor coordination and result in altered motor strategies becoming the norm (Lepley et al., 2015; Grooms and Myer, 2017). The *“control-chaos continuum”* proposes that the recovery sessions move from high control to high chaos, prescribing running loads under increasingly riskier conditions (Taberner et al., 2019). This is achieved by progressively incorporating greater perceptual and reactive neurocognitive challenges (Grooms and Myer, 2017; Taberner et al., 2019).

Lastly, it is important to frame RTS as a risk management endeavor with the risk being the inability to perform at equivalent or higher preinjury levels of performance. Injury risk management models in sports have been mostly established with a preventive mindset towards injury (van Mechelen et al., 1992; Roe et al., 2017), and already identified the need for an individualized approach (Roe et al., 2017). Therefore, a risk management perspective for RTS would allow the RTS stakeholders to reach decisions based on set criteria and lay the foundation to develop objective models of assessment. Such models could drive individualization on risk assessment and management.

## 5 Assessment of the return to sport progress

Evaluating the progress of RTS has proved equally challenging and the need for development and use of validated and reliable RTS assessment tools has been clearly stated (Ardern et al., 2016a; Losciale et al., 2019; Taberner, 2020; Marom et al., 2022). Marom and others demonstrated a high variability in defining, evaluating and reporting patterns of RTS after anterior cruciate ligament reconstruction (Marom et al., 2022). Patient reported outcomes provide a subjective way to assess the ongoing rehabilitation process and help understanding the level of readiness in athletes following injury (Ruzbarsky et al., 2018; Nelson et al., 2019; Marom et al., 2020). In comparison to validated patient reported outcomes (Ruzbarsky et al., 2018; Marom et al., 2020; Nelson et al., 2020; Hoenig et al., 2021) there is a lack of standardization and validation of proven RTS assessment tools (Marom et al., 2022). This limits the reliability, accuracy and overall comparability of RTS outcomes. Taken together with the high probability of reinjury in recovering athletes, it necessitates the development or usage of objective and reliable tools to accompany RTS stakeholders throughout the RTS spectrum.

Quantitative assessment of motor function has become increasingly important in clinical practice over the years. Functional assessments have been recently proposed (Hildebrandt et al., 2015) and validated (Herbst et al., 2015) in RTS after anterior cruciate ligament reconstruction, including stability tests, countermovement jumps, plyometric jumps and others (Barber-Westin and Noyes, 2011; Hildebrandt et al., 2015), however, without the use of standardized biomechanical equipment. Trasolini et al., proposed objective measurements using cameras in order to determine the range of motion in throwing athletes, during the RTS process (Trasolini et al., 2022). While this is undoubtedly a step in the right direction, such approaches have been rare and have mostly been used to test the progress of the rehabilitation without providing specific feedback on where possible limitations may arise. Further, the lack of normative, population-wide values hinders the interpretation of these outcomes.

## 6 Biofeedback-based return to sport: Perspectives and opportunities

The RTS research field has been continuously developing and expanding over the last years, but also faces several challenges ahead. Here, we identified unsuccessful load management and rehabilitation as major contributors to injury, a lack of consensus in the RTS terminology, presented the main frameworks that aim to guide RTS and the need for more objective and quantitative assessments. Below we argue that wider adoption and systematic use of biomechanical assessments-especially in functional settings (e.g., during running or throwing), could expand or even enhance existing RTS frameworks by enabling more rigorous quantitative assessments, which in turn could be of benefit to the overall RTS process.

Recent advances in sensor sciences have increased the opportunities for accurate, wireless and long-lasting capturing of data. Assessment methods originating from the field of biomechanics can objectively increase our understanding of healthy and pathological movement based on the analysis of kinematic (the study of the motion of bodies in space with respect to time) and kinetic (the study of the forces associated with motion) parameters (Winter 1987; Bartlett, 2007). Physiological signals pertaining the human neural circuit, such as, the electric activity of muscles and brain have also been captured to provide insights into movement (Bizzi et al., 2008; Gwin et al., 2011; Kesić and Spasić, 2016; Taborri et al., 2020). Equipment commonly used in biomechanical testing such as cameras, force plates, electromyography, wearable inertial sensors and others can be, hence, used during the RTS continuum for quantitative assessment and for detecting changes between measurement sessions in standardized settings (Pappas et al., 2016; Hainline et al., 2017). However, in RTS the biological recovery of the affected tissue must be accompanied with adequate functionality, which constitutes a much greater challenge.

Running, jumping and throwing, which are predominant in most athletic disciplines, are uniquely advantageous to be examined with biomechanical equipment from a functional perspective. Running analysis, due to movements being stereotyped and/or cyclic, can be streamlined to produce quick assessments and comparisons. Multiple force plates, cameras and wearable sensors can be used to capture large amounts of data in an on-the-field setting. Parameters, such as spatiotemporal variables, force profiles (e.g., the way forces are shaped during the time that the foot is in contact with the ground), a wide spectrum of kinematic variables (e.g., range of motion, vertical displacement) and the contribution to mechanical power from different joints have been often used to assess performance changes (Taylor, 1985; Arampatzis et al., 2000; Angelozzi et al., 2012; Santuz et al., 2016; Ekizos et al., 2021). It is therefore possible that these metrics would provide unique insights to the RTS process. However, their effectiveness and validity in the context of RTS has yet to be systematically examined. One limitation is the difficulty of direct quantitative comparisons with preinjury levels of performance. However, extending the usage of such metrics in routine preinjury baseline measurements, could enable comparisons during the recovery protocol, or normative values could be established and utilized, where applicable.

It has long been suggested that the ability to reliably quantify lower-extremity biomechanical variables during dynamic tasks could reveal mechanisms related to injury risk factors (Ford et al., 2007). Indeed, it has been shown that certain biomechanical profiles are associated with higher incidence of anterior cruciate ligament injury in high school female athletes (Pappas et al., 2016). A full-body approach to biomechanical assessment has been also suggested for the examination of pain in the elite athlete (Hainline et al., 2017). Interestingly, this has not been systematically examined in RTS settings. We argue that information of force and kinematic profiles (e.g., distinguishing the contribution and distribution of power in the involved joints during running) could be invaluable in identifying weaknesses in the recovering athlete and introducing specific exercises to alleviate them.

There is amounting evidence that load management, is a prominent mechanism which could be important not only to predict injury, but also to guide RTS (i.e., *acute to chronic load ratio*) (Blanch and Gabbett, 2016; Soligard et al., 2016; Eckard et al., 2018). However, a one-size-fits-all approach does not address the variability existing between injuries, players, competitive demands and response to loading. Rehabilitation and training practice as it approaches and enters the RTS has to be targeted and, thus, highly individualized. Timing is especially important, due to affecting load perception. Fitness levels, body composition and playing level often fluctuate with time (Jones et al., 2017). Together with age and history of injuries these characteristics have a significant impact on the perception and experienced stress by the body. All change constantly, are highly individual and can drastically affect the risk of injury (Ekstrand et al., 2011; Zwerver et al., 2011; Rogalski et al., 2013; Jones et al., 2017). These characteristics are equally influencing the athlete before, during and after an injury occurs and can, thus, affect the progress of the rehabilitation and the risk of reinjury. Kinetic and kinematic analyses could deliver information on how the applied force is distributed (e.g., using force plates during walking and running), identifying compensatory patterns (e.g., motion analysis in throwing tasks). Biomechanical assessments during functional movements could, thus, enable a highly customizable approach in terms of load management by monitoring and accounting for fluctuations in individual characteristics.

During RTS-besides increasing load, the human body has to constantly incorporate new information about its state (ongoing rehabilitation process) and navigate increasingly demanding tasks. Biological systems have the property of robustness, defined as the ability to maintain function despite internal and external perturbations (Kitano, 2004). Training in the presence of external perturbations improves muscle strength, stability, and balance performance (Hamed et al., 2018; Bohm et al., 2020; McCrum et al., 2022) and the progressive incorporation of highly variable, spontaneous, and unanticipated movements has been proposed in the context of RTS (Taberner et al., 2019). Human movement reveals the hallmark characteristics of complex systems (Mayer-Kress et al., 2006). The broad term “complexity” can be used to define the emergence of different (i.e., more or less complex) strategies (Dusing and Harbourne, 2010; Bisi and Stagni, 2018) to accomplish a specific motor task and enrich the training process. Maintaining functionality despite increased complexity is a challenge for the system and could induce beneficial neuromuscular adaptations. In this context, this approach could be beneficial to the recovery process of the elite athlete. Fine-tuning the range and amplitude of training complexity through objective biomechanical measurements means that this process could be more individualized, taking into account specific needs and performance goals.

**FIGURE 1:**
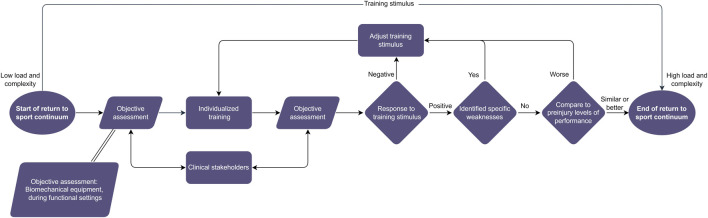
The biofeedback-based return to sport (RTS) approach. This approach places objective assessments at the core of the RTS process and applies the gathered information to inform it. The interaction with other RTS stakeholders should be emphasized. In the context of this paper objective assessments are meant as evaluations through biomechanical equipment utilized during functional settings.

We propose that biomechanical assessments would enable multiple biofeedback cycles, and, thus, a biofeedback-based approach in RTS. This approach could work independently, but also complimentary to existing RTS frameworks by providing quantitative and objective assessments. Further, it could facilitate interactions with the clinical stakeholders of the RTS process (e.g., identification of ankle instabilities through kinematic profiles or lower activation of specific muscles through electromyography), driving further clinical assessments. We argue that when employed to the RTS continuum, these biofeedback cycles could drive effective individualization. These assessments should aim to: a) identify weaknesses in kinetic and kinematic parameters, b) monitor load and complexity, c) compare current state of performance to inter- and intrapersonal values. Consequently, adjustments to the training program should be introduced. This approach accentuates the need for regular objective assessments of the athlete during the RTS continuum and the incorporation of this biofeedback to customize the load and complexity of the trainings (Figure 1).

Future studies should also consider a more holistic approach to RTS, by incorporating assessments of motor control. To maintain functionality during the execution of complex movements, humans rely on reflexes and sensory feedback to produce accurate, coordinated actions (Bernstein, 1967; Biewener and Daley, 2007; Grillner and El Manira, 2020). This is achieved through a modular interplay between muscles, sensory organs, and the central nervous system, which interact to produce meaningful, dynamically stable movement (Nishikawa et al., 2007). Knowing how body kinematics or muscle activation patterns behave during the production of simple repetitive activities such as locomotion is sufficient to understand the behavior of the system and quantify its ability to withstand perturbations (Dingwell and Cusumano, 2000; Ekizos et al., 2017; Santuz et al., 2018). Due to the nature of the information sought (i.e., related to motor control), these measurements often require a large number of repetitive movements. Athletic disciplines are, therefore, especially suitable since they require repetitive movement patterns or explosive movements executed with high frequency. We have recently demonstrated that measures of stability and analyses of muscle activation patterns are sufficiently accurate to confirm changes between speeds and conditions (Ekizos et al., 2018a; Santuz et al., 2018; Santuz and Akay, 2020), but also between different running techniques and adaptations to running training programs (Ekizos et al., 2017; 2018a). Such approaches are increasingly accessible (Santuz, 2022) and reliable (Santuz et al., 2017; Ekizos et al., 2018b; Fohrmann et al., 2022) and may prove valuable tools in assessing neuromuscular aspects pertaining to motor control after injury as well.

## 7 Conclusion

In the current manuscript we have seen that with high prevalence rates, injuries are part of elite sports and is often part of the athlete’s career in multiple ways. Further, that the management of the period after the injury is crucial for an effective RTS. There is a need for objective and reliable RTS assessment tools in order to encourage more quantitative assessments. In the ongoing discussion of how to improve the RTS process, we argue that biofeedback cycles using objective assessments could drive an effective and individualised RTS. The incorporation of biomechanical parameters derived during functional settings seems ideal for this purpose. The development of regular standardized measurement procedures could enhance the applicability of this approach by establishing baseline and normative values.

## Data Availability

The original contributions presented in the study are included in the article/supplementary material, further inquiries can be directed to the corresponding authors.
